# Sarcopenic obesity in cancer

**DOI:** 10.2478/raon-2024-0011

**Published:** 2024-02-21

**Authors:** Mihaela Jurdana, Maja Cemazar

**Affiliations:** Faculty of Health Sciences, University of Primorska, Izola, Slovenia; Institute of Oncology Ljubljana, Ljubljana, Slovenia

**Keywords:** sarcopenia, obesity, cancer, inflammation

## Abstract

**Background:**

Sarcopenic obesity is a relatively new term. It is a clinical condition characterized by sarcopenia (loss of muscle mass and function) and obesity (increase in fat mass) that mainly affects older adults. As the incidence of sarcopenia and obesity increases worldwide, sarcopenic obesity is becoming a greater problem also in cancer patients. In fact, sarcopenic obesity is associated with poorer treatment outcomes, longer hospital stays, physical disability, and shorter survival in several cancers. Oxidative stress, lipotoxicity, and systemic inflammation, as well as altered expression of skeletal muscle anti-inflammatory myokines in sarcopenic obesity, are also associated with carcinogenesis.

**Conclusions:**

Reported prevalence of sarcopenic obesity in cancer varies because of heterogeneity in definitions and variability in diagnostic criteria used to estimate the prevalence of sarcopenia and obesity. Therefore, the aim of this review is to describe the definitions, prevalence, and diagnostic criteria as well as the mechanisms that cancer has in common with sarcopenic obesity.

## Introduction

Sarcopenic obesity is a clinical condition characterised by the coexistence of obesity, excess fat mass (FM) and sarcopenia (decrease in skeletal muscle mass and function).^1,2^ Baumgartner was the first to propose the term sarcopenic obesity, which is considered a unique clinical condition distinct from obesity and sarcopenia alone.^1^ The prevalence of sarcopenic obesity is expected to become a public health problem as the prevalence of sarcopenic obesity in adults is rapidly increasing worldwide. The clinical consequences of sarcopenic obesity are considerably more severe than those of sarcopenia or obesity alone. In comparison to both, it can lead to physical disability, morbidity, and even mortality.^3^ In addition, cancer is another serious global health problem with increasing incidence and mortality worldwide.^4^ Interestingly, sarcopenic obesity is more common in older adult cancer patients and patients with other chronic diseases, but its prevalence is also increased in younger patients with obesity and chronic diseases such as cancer and is associated with worse treatment outcomes.^5^ Sarcopenic obesity and cancer, as well as other chronic diseases, share some key pathogenetic mechanism such as inflammation, oxidative stress, and insulin resistance, which are considered key factors.^6,7,8^ Among these, insulin resistance is considered a central condition in both, cancer and sarcopenic obesity.^8^

Several factors in sarcopenic obesity may lead to progressive loss of muscle mass and altered fat metabolism, which influence each other in a reciprocal pattern. Sarcopenia is known to be a common problem in cancer patients, especially if they are suffering cachexia and is associated with physical disability, surgical complications, increased risk of severe toxicity during cancer treatment, prolonged hospitalisation, and shortened survival.^6^ Sarcopenia can occur at any stage of cancer and in any body mass index (BMI) category and is often associated with obesity. In addition, obesity, particularly abdominal obesity, can independently lead to loss of muscle mass and function due to the negative effects of oxidative stress, inflammation, and insulin resistance, all of which negatively impact muscle mass.^7^ Recently, there has been an increasing interest in utilizing body composition phenotype as an additional indicator of cancer prognosis. Because the burden of sarcopenia and obesity are concurrent, they can be expected to have a combined impact on health outcomes in several clinical settings, including cancer.^6^

As the prevalence of sarcopenic obesity with poor prognosis is rapidly increasing in cancer patients, in this review we discuss definitions, prevalence, mechanisms and management strategies for sarcopenic obese patients in oncology. This review aims to provide clinician’s with additional evidence helping them to make rational decisions that will improve patients’ outcome.

## Identification of sarcopenic obesity and diagnostic criteria

Sarcopenic obesity has been identified using various definitions and diagnostic criteria. Screening for sarcopenic obesity is based on the simultaneous presence of increased BMI or waist circumference with ethnicity-specific cut-offs and surrogate parameters of sarcopenia (clinical symptoms, validated questionnaires -SARC-F in the elderly). In addition, altered body composition is required to make a definitive diagnosis.^9,10^

Many studies have investigated the prevalence of sarcopenic obesity in different cohorts of cancer patients, including oropharyngeal^11^, lung^12,13^, gastrointestinal^14,15,16,17,18,19,20,21,22,23,24^, liver and pancreatic^25,26^, urinary^27,28,29^, breast^30^, melanoma^31^ and lymphoma.^32^ On the other hand, the lack of uniform diagnostic criteria for sarcopenic obesity hampers the identification of patients and the assessment of associated outcomes and, consequently, negatively affects the development of prevention and treatment strategies for sarcopenic obesity. The prevalence of sarcopenic obesity in studies that include cancer patients varies from 1–29% in studies of patients in all BMI categories and from 15% to 36% in studies of overweight/obese patients.^6^ The lowest prevalence of sarcopenic obesity is found in early disease stages and the highest in locally advanced or metastatic disease.^6^ Heterogeneity in diagnostic criteria between studies, as well as the metabolic impact of different cancer types, other patient characteristics such as ethnicity, and concurrent comorbidities, limit interpretation of results. In addition, The European Society for Clinical Nutrition and Metabolism (ESPEN) and the European Association for the Study of Obesity (EASO) confirmed heterogeneity in definition and diagnostic approaches for sarcopenic obesity, due to different definition of obesity and sarcopenia, differences in methodologies to assess body composition and function, as well as in the applied references values for variables used.^9^ Regardless of this variability, most studies report that sarcopenic obesity is an important problem in cancer patients and a negative prognostic factor.

## Commonly used technics in oncology setting

Various techniques and body composition parameters with different cut off as well as muscle function parameters have been used to identify sarcopenic obesity. Body composition is traditionally measured using Dual Energy X-ray Absorptiometry (DEXA) or Bioelectrical Impedance Analysis (BIA) but is known to have some limitations.^9^ Currently, computed tomography (CT) and magnetic resonance imaging (MRI) are considered the gold standard methods. Both CT and MRI are not routinely used to assess sarcopenia and obesity due to high costs and radiation concerns for CT.^33,34^ CT should be used when possible (e.g., in patients undergoing a scan for diagnostic reasons in oncology).^9^ In cancer patients, the CT scan provides the highest available precision in determining body composition parameters by measuring the cross-sectional area of total skeletal muscle at the third lumbar vertebra (L3), which correlates strongly with total body skeletal muscle mass.^35^

The DEXA method, which is considered inexpensive, is the most accurate method for measuring appendicular muscle mass, and exposes the patient to minimal radiation, but is not widely available. The BIA method is an alternative, inexpensive, and readily available method, but results are easily confounded by various factors, especially fluid status.^34^ All these techniques provide anatomic information about the patient but not functional information, because loss of muscle function is also required to diagnose sarcopenia.^36^ Therefore, the diagnostic process must include a direct assessment of altered skeletal muscle function parameters along with altered body composition. Thereafter, individuals with a positive diagnosis should be classified into two stages: Stage I, when no clinical complications are present, and Stage II, with clinical complications such as chronic disease (e.g. cancer), dietary events (weight loss, decreased food intake), immobility, falls, and other complaints associated with altered body composition and muscle function.^9^ All procedures are summarized in Table 1.

**TABLE 1.** Selected criteria to identify sarcopenic obesity. Altered skeletal muscle function parameters considering muscle strength and physical performance and altered body composition parameters should be present to assess sarcopenic obesity

**SCREENING**	**DIAGNOSIS**	**STAGES**
High BMI and WC (based on ethnic cut-points)	Altered skeletal muscle strength (HGS, chair stand test)	STAGE 1: Without complications
Surrogate markers of sarcopenia: (clinical symptoms or validated questionaries’ e.g. SARC-F)	Altered body composition (increased FM, decrease MM)	STAGE 2: One or more complications attributable to sarcopenic obesity

BMI = body mass index; FM = fat mass; HGS = hand grip strength; MM = muscle mass; SARC-F = strength, assistance in walking, rise from a chair, climb stairs, and falls; WC = waist circumference;

## Biological pathways leading to sarcopenic obesity

The biological pathway leading to sarcopenic obesity includes changes in body composition related to ageing, hormonal changes, the interplay between metabolism and inflammation, environmental factors such as poor nutrition and lack of exercise and chronic diseases.^37,38,39,40^ This results in a decrease in oxidative capacities, mitochondrial number, atrophy of fast type II muscle fibres and neurodegeneration, decrease in protein synthesis and increase muscle protein degradation.^41^

### Metabolic disfunctions

Both, skeletal muscle mass and adipose tissue interplay with several cancers at metabolic levels.^42,43,44,45^ Few studies have investigated the pathway between sarcopenia, obesity and cancer. The mechanisms involved in pathogenesis of metabolic imbalances associated with obesity are in part common with pathway modulating cancer related sarcopenia.^5^ Inflammation promoted by cancer and/or inadequate intake of essential nutrients could contribute to the presence of fatigue and decrease in physical activity and mobility of cancer patients.^6^

Physical inactivity can lead to skeletal muscle loss by reduced protein anabolic pathway and activation of proteolytic pathway.^5,6^ In addition, inadequate food intake can also impair muscle anabolic pathway due to low omega −3 (n-3) fatty acid intake. Namely, it was demonstrated that low level of n-3 fatty acids is associated with loss of muscle mass and skeletal muscle fat infiltration or myosteatosys.^46^ In addition, atrophy of fast type II muscle fibre and switch to slow type I muscle fibre increase the lipid deposition into the muscle.^6^ Importantly, myosteatosis is associated with metabolic muscle disfunction and muscle function loss and is widespread in cancer associated mal-nourished patients.^47,48^ A vicious cycle between myocytes and adipocytes is responsible for sarcopenic obesity. Specifically, adipose tissue inflammation and dysfunction leads to overproduction of fatty acids which in combination with low oxidation capacity of skeletal muscle stimulate the formation of intramyocellular lipid (IMCL).^49^ This process blocks the translocation of glucose transporter type 4 (GLUT4) to the surface of the muscle fiber and therefore hampers uptake of glucose by skeletal muscle resulting in a decrease of glucose utilization and increase of fatty acid oxidation in mitochondria, which leads to impaired insulin sensitivity of skeletal muscle, inhibition of mitochondrial respiration, increase in reactive oxygen species formation (ROS), myocyte toxicity, inflammation and finally sarcopenia.^5,6^ Various forms of fat accumulation in skeletal muscle have been associated with insulin resistance, mitochondrial dysfunction and decreased muscle contracting force.^50^ Based on these mechanisms, myosteatosis is a potentially important factor in sarcopenic obesity in the cancer setting, which could contribute to further muscle dysfunction in sarcopenic obese cancer patients and is an independent predictor of reduced survival in cancer patients.^47,51^ Additionally, ectopic fat deposition surrounding muscle accelerate proteolysis in muscle tissue leading to further muscle loss and worsen outcome.^47^

Another consequence of insulin resistance is the reduction in the uptake of amino acids by muscle cells, altering the balance of protein synthesis/degradation in favour of proteolysis. It is proposed that the amino acids released by muscle proteolysis in sarcopenia contribute to the supply of tumor growth.^8^

### Hormonal imbalances

Furthermore, cancer is associated with an alteration in hormones that severely affects skeletal muscle and fat metabolism.^6^ The body composition of cancer patients is associated with insulin, insulin resistance, and the hormone ghrelin, the levels of which are modulated in obese patients and affect skeletal muscle metabolism. In addition, increased levels of stress hormones and a decrease in androgens and estrogens affect the anabolic and catabolic conditions for muscle protein metabolism and lead to alterations in the production and metabolism of anabolic hormones (growth hormone, insulin-like growth factor (IGF-1)) that may result in a sarcopenic obese phenotype.^52,53^ Increasing insulin resistance is associated with an increase in intramyocellular fat mass and loss of muscle function.^54^

### Cytokine imbalances

The systemic inflammatory condition in sarcopenic obesity as well as the altered expression of myokines and adipokines are also involved in carcinogenesis. Skeletal muscle and adipose tissue are considered endocrine organs due to release anti-inflammatory myokines and pro-inflammatory cytokines, respectively.^55^ Myokines are proteins released by muscle cells in response to contractions. They play autocrine, paracrine, and endocrine roles in many exercise-induced adaptations (e.g., muscle hypertrophy and cancer protection).^55,56^ The release of pro- and anti-inflammatory cytokines by adipose and muscle tissue has a strong influence on skeletal muscle and adipose tissue metabolism and is involved in various cancer related changes in body composition.^5^ Dysregulation of pro- and anti-inflammatory cytokines is responsible for muscle intracellular adipose tissue. They contribute to the secretion of myostatin a negative regulator of muscle mass secreted by skeletal muscle cells, tumor necrosis factor −α (TNF-α), proinflammatory interleukin −6 (IL-6), interleukin −1β (IL-1β), mononuclear chemoattractant protein-1 (MCP-1), and downregulate the secretion of anti-inflammatory adiponectin, leading to lipotoxicity and insulin resistance.^8,37,57,58,59^ Inflammatory cytokines directly affect skeletal muscle and accelerate muscle proteins degradation and apoptosis and induces muscle tissue reduction and fat tissue accumulation.^5,6,8^ Levels of IL −6 and TNF-α are further increased by the hormone leptin, reducing the anabolic pathway of IGF-1 and enhancing lipotoxicity. The cytokine-like hormone leptin is a classic adipokine that is secreted by adipocytes, its blood concentration correlates with triglyceride accumulation in adipocytes.^60^ In addition to this, the impaired secretion of skeletal muscle anti-inflammatory myokines such as interleukin −15 (IL-15), irisin, muscle derived IL-6 accelerate muscle atrophy and disfunction.^8,56,59,61^

All these cytokines are known to be involved in several cancer induced alteration of body composition.^62^ Altered fat metabolism promotes inflammation, having an important role in cancer and non-cancer muscle wasting^63^ creating a vicious cycle for sarcopenic obesity, leading to morbidity and mortality.

**FIGURE 1.**
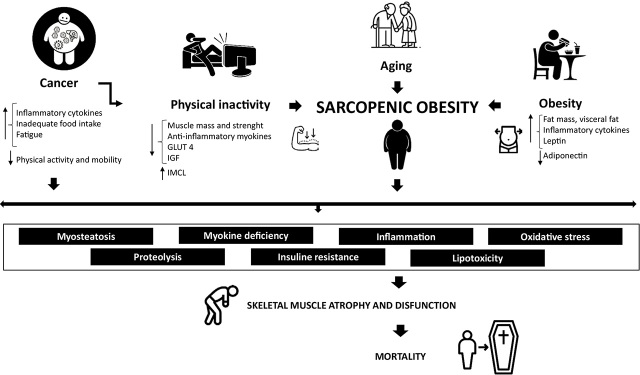
Main pathophysiological mechanisms in cancer patients with sarcopenic obesity. Body composition (low skeletal muscle mass and strength, increase in fat mass), inflammation, insulin resistance, myosteatosis, myokine dysregulation, and oxidative stress in sarcopenic obese cancer patients significantly induce muscle proteolysis, leading to muscle wasting and dysfunction and premature mortality.

Sarcopenic obesity and carcinogenesis, therefore, are mediated by mechanisms, such as insulin resistance, adiposity, proteolysis, myosteatosis, inflammation, oxidative stress, imbalance of adipokines and myokines (Figure 1).

## Therapeutic strategies

Patients with cancer and sarcopenic obesity have several specific adverse clinical outcomes, including higher risk of dose-limiting toxicity, surgical complications, prolonged hospitalisation, physical disability, and shorter survival. This was demonstrated for several cancers.^6^ Chemotherapy has been shown to alter body composition by reducing lean mass, thus favouring the development of sarcopenic obesity.^64^ Low muscle mass and strength is common in any cancer stage and is clearly considered an independent predictor for cancer progression, making it a preferred target in the treatment of sarcopenic obesity. On the other hand, obesity is not univocally associated with worse outcome in cancer patients regardless of the cancer type.^65^ Although obesity is considered an important risk factor for the development of various cancers, the presence of moderate obesity paradoxically appears to be a protective factor that may improve response to treatment and consequently survival in patients but there are conflicting and controversial results.^66^

Several therapeutic strategies, exercise and nutritional exists to counteract sarcopenic obesity. Prado *et al.* have shown that poor nutritional status in cancer is primarily manifested by a severe loss of muscle mass, which can occur at any stage (from curative to palliative treatment) and often co-exists with obesity. They have addressed the role of diet in preventing and reversing sarcopenia in cancer patients, which may also apply to sarcopenic obesity.^67^ Nutritional strategies comprise the importance of adequate intake of macro- and micronutrients, including high-quality proteins, branched-chain amino acids (leucine), β-hydroxy-β-methylbutyrate, glutamine, carnitine, creatine, fish oil/eicosapentanoic acid (EPA and DHA), vitamins/minerals (e.g., vitamin D), and multimodal approaches (diet, exercise, and medications) to counteract low muscle mass in cancer.

Moreover, physical activity could be an important and effective treatment strategy to reverse sarcopenia by promoting insulin sensitivity, reducing oxidative stress and inflammation, and stimulating mitochondrial biosynthesis. Both resistance training and aerobic exercise have been shown to improve muscle mass and physical performance.^68,69^ We note that standard treatment recommendations derived from studies in the elderly and certain diseases may not be applicable to cancer patients due to fatigue or pain. However, many intervention studies of physical activity in cancer indicate some benefit of exercise for muscle strength and endurance.^70^ In addition, cancer obese individuals, who exercise more and are not insulin resistant or hypertensive might then have a lower mortality risk.^8^

Another effective treatment for sarcopenic obesity in older adults and cancer patients is skeletal muscle electrostimulation. It causes contraction of muscle fibres via neuromuscular activation and can induce a change in body composition.^71^ Whole-body vibration therapy has been shown to significantly increase muscle strength and function in older adults.^72^ Further clinical studies are needed to verify its efficacy in clinical practise.

## Conclusions

The prevalence of sarcopenic obesity is considered a novel factor of great clinical relevance in cancer patients, leading to postoperative complications, worse functional status, and shorter survival possibly mediated by interactions among pathophysiological mechanisms (inflammation, insulin resistance, dysregulation of myokines and pro-inflammatory cytokines). Specific prevention and treatment strategies are needed to address sarcopenic obesity in cancer patients. One of the major challenges in prevention strategies is to maintain skeletal muscle mass and function and reduce fat mass, because the combination of decreasing skeletal muscle mass and increasing fat mass leads to physical limitations that worsen the prognosis for chronic disease, including cancer. Exercise and proper nutrition are two key components in the prevention and treatment of sarcopenic obesity, but effective interventions should be explored for cancer patients.^3,4,6,56,73^ The key question is how to maintain muscle anabolism in an energy deficit situation to avoid a high percentage of weight loss in the form of lean mass in this muscle loss prone population.

The underestimated prevalence of sarcopenic obesity in cancer are the consequence of the lack of standard methods and definitions used in previous studies. Therefore, further studies need to focus on screening sarcopenic obesity in cancer patients, and additional studies are needed to clarify the pathogenesis of sarcopenic obesity, with emphasis on identifying new markers.

In conclusion, the development of standardised diagnostic criteria is urgently needed. Up to now the identification of sarcopenia by measuring muscle mass and strength as a physical function has been done only in few studies, while in all others muscle mass was used as the only criteria.

ESPEN and EASO have launched an initiative to reach expert consensus on diagnostic procedures that include an assessment of skeletal muscle function followed by an assessment of body composition. Individuals with sarcopenic obesity should be classified into stages 1 and 2 based on clinical complications associated with body composition or muscle dysfunction. The proposed definition should be implemented in routine clinical practice. In addition, validation and prospective follow-up studies and secondary analyses of existing cohorts are proposed and encouraged by ESPEN and EASO.^9^
